# *Capparis sepiaria*-Loaded Sodium Alginate Single- and Double-Layer Membrane Composites for Wound Healing

**DOI:** 10.3390/pharmaceutics16101313

**Published:** 2024-10-10

**Authors:** Sindi P. Ndlovu, Keolebogile S. C. M. Motaung, Mapula Razwinani, Sibusiso Alven, Samson A. Adeyemi, Philemon N. Ubanako, Lindokuhle M. Ngema, Thierry Y. Fonkui, Derek T. Ndinteh, Pradeep Kumar, Yahya E. Choonara, Blessing A. Aderibigbe

**Affiliations:** 1 Department of Chemistry, University of Fort Hare, Alice Campus, Alice 5700, South Africa; 201304407@ufh.ac.za; 2 Global Health Biotech Pty Ltd., Pretoria 0087, South Africa; keo@globalhealthbiotech.co.za; 3 Department of Biotechnology and Food Science, Faculty of Applied Sciences, Durban University of Technology, Durban 4000, South Africa; nomphar@yahoo.com; 4 Department of Chemistry, Nelson Mandela University, Gqeberha 6001, South Africa; s217616712@mandela.ac.za; 5 Wits Advanced Drug Delivery Platform Research Unit, Department of Pharmacy and Pharmacology, School of Therapeutic Science, Faculty of Health Sciences, University of the Witwatersrand, Johannesburg 2193, South Africa; samson.adeyemi@wits.ac.za (S.A.A.); philemon.ubanako@wits.ac.za (P.N.U.); 845407@students.wits.ac.za (L.M.N.); pradeep.kumar@wits.ac.za (P.K.); yahya.choonara@wits.ac.za (Y.E.C.); 6 Department of Biotechnology and Food Technology, Faculty of Science, University of Johannesburg, Doornfontein Campus, Johannesburg 2028, South Africa; youmbifonkui@yahoo.com; 7 Drug Discovery and Smart Molecules Research Labs, Centre for Natural Product Research, Department of Chemical Sciences, University of Johannesburg, Doornfontein Campus, Johannesburg 2028, South Africa; dndinteh@uj.ac.za

**Keywords:** wound dressing, *Capparis sepiaria* extract, wounds, sodium alginate, polyvinyl alcohol, chronic wounds, propylene glycol, gum acacia, Pluronic F127, and membranes

## Abstract

**Background**: Effective wound dressing is the key solution to combating the increased death rate and prolonged hospital stay common to patients with wounds. **Methods**: Sodium alginate-based single- and double-layer membranes incorporated with *Capparis sepiaria* root extract were designed using the solvent-casting method from a combination of polyvinyl alcohol (PVA), Pluronic F127 (PF127), and gum acacia. **Results**: The successful preparation of the membranes and loading of the extract were confirmed using Fourier transform infrared spectroscopy (FTIR) and X-ray diffraction (XRD). The prepared membranes were biodegradable and non-toxic to human skin cells (HaCaT), with high biocompatibility of 92 to 112% cell viability and good hemocompatibility with absorbance ranging from 0.17 to 0.30. The membrane’s highest water vapor transmission rate was 1654.7333 ± 0.736 g/m^2^/day and the highest % porosity was 76%. The membranes supported cellular adhesion and migration, with the highest closure being 68% after 4 days compared with the commercial wound dressings. This membrane exhibited enhanced antimicrobial activity against the pathogens responsible for wound infections. **Conclusions**: The distinct features of the membranes make them promising wound dressings for treating infected wounds.

## 1. Introduction

Wounds are a significant global health challenge that often lead to morbidity and mortality [1,2,3]. They are ranked the fourth most common cause of trauma worldwide [4]. Every year, thousands of deaths are caused by chronic wounds in third-world countries. The healing process of wounds is usually prolonged during the inflammation phase owing to the invasion of resistant bacterial pathogens, leading to the delayed deposition of collagen [5,6,7]. The complexity of skin injuries necessitates advanced wound dressings to promote accelerated healing, thereby preventing the invasion of microbes and restoring the skin’s function [1,8,9]. Wound dressings are designed to protect the skin to further restore the integrity of the outer layer (epidermal tissue) and the inner layer (dermal tissue) of the damaged skin tissue [10,11]. An effective wound dressing should be biocompatible and non-toxic, absorb excess exudate, be biodegradable, have antibacterial potency and mechanical properties, control drug release, and promote keratinocyte and fibroblast proliferation [12]. Moistened wound dressings are most desired due to their high oxygen permeability and water content properties that keep the surface of the wound moist, facilitating epithelialization and rapid healing [13].

The preparation of an ideal wound dressing with antibacterial efficacy, limited side effects, and non-allergic reactions remains a major challenge [14,15]. Despite the challenges encountered, wound dressing should address the patient’s needs, prevent complications, and facilitate the wound-healing process [16]. In this study, membrane wound dressings were prepared from biodegradable synthetic polymers and natural polymers for enhanced wound-healing properties [17,18]. Membranes are flexible wound dressings that provide a multifaceted approach to wound care, offer protection, promote healing, and prevent infections. The exploration of various synthetic and natural materials, together with the application of technologies to develop wound dressings, has led to wound dressings that are not only effective in managing wounds but also compatible with the body’s natural healing processes [19,20]. Membranes provide a protective barrier against microbial penetration, maintain a moist healing environment, allow for gas exchange, and exhibit a high surface area and porosity [21]. Membranes can be designed as single or double layer with distinct properties. The difference between the single- and bilayer membranes lies in their structural complexities and functionalities. Bilayer membranes offer advantages, including enhanced mechanical strength, high stability, and increased porosity and adsorption capacity, and are effective as skin substitutes to promote wound healing and the regeneration of skin tissue and sustain prolonged drug release. The bilayer structures can be tailored to meet specific performance criteria, making them versatile and effective wound dressings [22,23]. In this study, sodium alginate (SA) was explored for the design of the membranes.

SA is a biopolymer derived from brown seaweed and it is frequently used to prepare wound dressings because of its biocompatibility, biodegradability, ability to absorb excess wound exudates, and ability to maintain a moist healing environment [8,12,24,25]. Plant extracts have also been reported to promote skin regeneration and accelerate the wound healing process owing to their antioxidant, antibacterial, and anti-inflammatory properties. In addition, plant extracts contain phytochemicals that play a significant role in wound healing [26,27,28,29].

Recent studies have focused on enhancing the therapeutic efficacy of SA-based wound dressings by incorporating plant extracts. SA-based hydrogel films loaded with *Betula utilis* bark extract demonstrated antibacterial activity and high wound contraction in animal models [30]. Similarly, a nanofibrous mat of polyvinyl alcohol/alginate incorporated with *Arnebia Euchroma* extract showed excellent wound healing with high collagen synthesis and re-epithelization [26]. These findings suggest that encapsulating plant extracts into sodium alginate-based wound dressings can significantly improve wound healing outcomes. Sodium alginate-based wound dressings are important in wound management. Incorporating plant extracts into these dressings has emerged as a promising strategy to enhance their healing properties, as evidenced by the antibacterial and wound-healing efficacy demonstrated in the studies above.

In this study, sodium alginate-based single- and double-layer membranes were fabricated and incorporated with *Capparis sepiaria* root extract to treat infected wounds. The membranes were fabricated using a solvent-casting method and characterized to evaluate their suitable use as wound dressings. The wound-healing efficacy of the *Capparis sepiaria* root plant extract was evaluated. The morphological, physicochemical, and mechanical properties of the membranes were evaluated. The biodegradability, biocompatibility, exudate absorption capacity, oxygen permeability, hemostatic effect, wound closure rate, and antibacterial efficacy of the membranes were evaluated to validate them as potential wound dressings.

## 2. Materials and Methods

### 2.1. Reagents

All the membranes were prepared using distilled water. Sodium alginate (SA), polyvinyl alcohol (PVA), and Pluronic F127 (PF127) were purchased from Sigma-Aldrich (Johannesbug, South Africa). Propylene glycol, sodium dihydrogen phosphate, sodium hydroxide, and gum acacia were purchased from Merck Chemicals (Johannesburg, South Africa). *Capparis sepiaria* root plant extract was supplied by Global Health Biotech (Pty), Ltd., Pretoria, South Africa. The reagents were used as received without further purification.

### 2.2. Experimental

#### 2.2.1. Preparation of the Single-Layer Membranes (SLMs)

The SLMs were fabricated using a solvent-casting method ((a) in Table 1). Varying quantities of SA and gum acacia were dissolved in 5 mL of distilled water at room temperature with constant stirring. PVA and PF127 solutions were prepared by dissolving them in 5 mL of distilled water at 80 °C and ice water, respectively, with continuous stirring. After the four solutions were successfully prepared, the SA solution was added dropwise to the PF127 solution, followed by adding gum acacia and PVA solutions while stirring on a magnetic stirrer. PF127 was used as a surfactant and polymer. It is also characterized by excellent biodegradability and biocompatibility. PEG was used as a skin enhancer to increase drug permeation through the skin. A 4 mL propylene glycol solution containing *Capparis sepiaria* root extract was added to the resulting polymer blend and stirred to obtain a uniform polymer mixture. The obtained polymer mixture was cast on a Petri dish and dried at 50 °C overnight. The obtained membranes were stored in a desiccator.

#### 2.2.2. Preparation of Double-Layer Membranes (DLMs)

The DLMs comprised a backing and top layers, both of which were prepared separately. The layers were fabricated using a solvent-casting method. The backing layers for all DLMs were prepared by dissolving 2.4 g of PVA in distilled water (20 mL) at 80 °C with stirring until a uniform solution was obtained. The uniform PVA mixture was poured into a Petri dish and left at room temperature for 24 h. After 24 h, the top layer was prepared using the same method reported for SLMs in Section 2.2.1. and their composition is shown in (b) in Table 1. The obtained polymer mixture was poured on top of the backing layer and dried at 50 °C in an oven overnight. The obtained membranes were further characterized.

#### 2.2.3. Surface pH of the SLM and DLM Wound Dressings

The surface pH of the membranes was evaluated by mimicking the actual wound model reported by Tenorová et al. and Vinklárková et al. [31,32]. The membranes were submerged in a buffer solution of pH 7.4. The membranes were submerged in 20 mL of buffer solution simulating wound exudate and covered with a lid to prevent liquid evaporation. After 24 h, the pH of the membranes in the wound model was evaluated and measured in triplicate using the pH meter BASIC 20+ (Barcelona, Spain).

### 2.3. Characterization

#### 2.3.1. FTIR

FTIR was performed on *Capparis sepiaria* root extract together with the SLMs and DLMs to confirm the successful preparation of the membranes and the loading of the plant extract. It was performed on a Perkin Elmer Spectrum 100 spectrometer (Waltham, MA, USA) from 4000 to 500 cm^−1^ and was plotted using Origin software, ORIGINPRO^®^2023b. The membranes were cut to fit the diamond crystal, and the maximum force was applied via clamping to obtain the FTIR spectrum.

#### 2.3.2. SEM

SEM analysis was performed to study the surface morphology of the prepared membranes. Samples of SLMs or DLMs were coated with gold prior to analysis. SEM analysis was performed at 15 kV on a JSM-6390LV microscope (Tokyo, Japan).

#### 2.3.3. AFM

AFM was performed to evaluate the surface roughness of the prepared membranes. It was performed on a Veeco atomic force microscope to obtain information on the surface roughness (R_a_) and root mean square roughness (R_q_) of the membranes.

#### 2.3.4. Porosity

Following the SEM results, the porosity was measured to evaluate the porous nature of the prepared membranes. The porosities of the prepared membranes were determined using the liquid-displacement method. Membranes of equal mass were submerged in 2 mL of ethanol and weighed after 1, 2, 3, and 24 h. The % porosity was calculated following the methods reported by Ghanbari et al. [33] and Nirmla et al. [34], and the equation is as follows:

(1) $$\mathrm{Porosity}\;\%=\frac{\left(Wb-Wa-Wc\right)}{\left(Wb-Wc\right)}\times 100\%$$

where ***Wa*** is the initial weight of the membrane before immersion in the ethanol medium, ***Wb*** is the weight of the ethanol and the membrane after being submerged in ethanol, and ***Wc*** is the weight of the ethanol after removing the membrane.

#### 2.3.5. WVTR

WVTR analysis of the membranes (i.e., SLMs and DLMs) and control (commercial wound dressing) was carried out according to the American Society for Testing and Materials (ASTM) method [35,36]. A sample vial (15 mL, with a diameter of 0.8 cm) was employed for the analysis, and the membranes were cut to cover the circular opening of the sample vial of 1.6 cm radius. The sample vial was filled with 5 mL of distilled water, and the membrane was mouthed and sealed on the sample vial opening using Parafilm. The entire setup was weighed, and the initial mass was recorded. After the setup was weighed, it was placed in a warm bath shaker set to 37 °C for 24 h for the membranes and controls. After 24 h, the setup was weighed again, and the WVTR was calculated using Equation (2):

(2) $$\mathrm{WVTR}=\frac{W_{B-W_{A24h}}}{A}\times \;10^{6}$$

***W_B_*** is the initial weight of the entire setup, ***W_A24h_*** is the weight after 24 h, and ***A*** is the area of the circular opening of the sample vial.

#### 2.3.6. Swelling Behavior

The swelling behavior of the membranes was determined according to previously reported procedures. The swelling behavior of the membranes was evaluated by weighing the wound dressings and submerging them in a buffer solution of pH 7.4, mimicking chronic wound exudate [37,38]. The membranes were removed at predetermined intervals of 1, 3, and 24 h. The excess buffer on the surface of the membrane was removed by gently wiping the surface of the wound dressing with filter paper. The swelling behavior percentage was calculated using Equation (3):

(3) $$\mathrm{Swelling}\;\%=\frac{Ma-Mb}{Mb}\times 100\%$$

where ***Ma*** is the weight of the membrane after immersion in the buffer solution, and ***Mb*** is the initial weight of the membrane before immersion in the buffer solution.

#### 2.3.7. XRD

XRD analysis was conducted to determine the presence of free drugs in the membranes. The membranes were finely ground, and the analysis was performed on a PANalytical X’pert PRO (Eindhoven, The Netherlands) fitted with a Cu Kα (λ = 0.154 nm) radiation source with a voltage of 45 kV and a current of 40 mA. All the powdered membrane samples were mounted on sample holders and scanned. The diffraction peaks were then changed to the d-spacing, permitting material identification. The two theta values ranged from 5 to 90 degrees.

#### 2.3.8. Biodegradation Studies

In vitro biodegradation studies of the membranes (SLMs and DLMs) were performed following a previously reported procedure [39,40]. A biodegradation analysis was performed to evaluate the degradability of the membranes under physiological conditions. The degradation of the membranes was studied in a phosphate-buffered (PBS) solution of pH 7.4, mimicking the pH of chronic wound exudate. The membranes were weighed, immersed in a buffer solution (pH 7.4), and incubated in a warm bath at 37 °C for 3 weeks. After 1, 2, and 3 weeks, the membrane was removed from the buffer solution, rinsed several times with distilled water, and dried in an oven at 50 °C. The degradation of the prepared membranes was calculated using Equation (4):

(4) $$\mathrm{Degradation}\;(\%)=\frac{M_{B}-M_{t}}{M_{B}}\times 100\%$$

***M_B_*** is the initial weight of the membranes before incubation, and ***M_t_*** is the weight after 1, 2, and 3 weeks of incubation and oven drying, respectively.

#### 2.3.9. Mechanical Properties

The mechanical properties of the membranes were evaluated to determine their flexibility and stiffness. The mechanical properties of the membranes, such as tensile strength (TS) and elongation at break, were determined using the D 882-18 Standard [41] test method for the tensile properties of thin sheets. The samples were cut into rectangular strips of the dimensions 7 × 30 mm. The ends of the strips were placed between the cardboard grips using double-sided adhesive tape. The membranes were conditioned for 24 h at 20 ± 2 °C and 65% ± 2% RH. The crosshead speed was 10 mm/min for the various cell loads. All data were analyzed in triplicate.

#### 2.3.10. In Vitro Antibacterial Analysis

An in vitro antibacterial analysis of the membranes was performed to determine the minimum inhibitory concentration (MIC) following the procedure reported by Fonkui et al. [42]. The membranes were dissolved to obtain a stock concentration of 1 mg/mL. The obtained solutions were then serially diluted 6 times into a nutrient broth of 100 µL in 96 well plates to concentrations of 500, 250, 125, 62.5, 31.25, and 15.625 µg/mL. The 100 µL of the obtained solutions were seeded and duplicated with overnight bacterial culture of 100 µL, which was brought to 0.5 Mc Farland in broth nutrient. Streptomycin, *Capparis sepiaria* plant root extract, ampicillin, the control (commercial wound dressing), and nalidixic acid were used as positive controls, and the negative controls contained nutrient broth at 50% in DMSO.

#### 2.3.11. In Vitro Cell Viability

The in vitro cytotoxicity of the membranes was evaluated using the MTT assay to assess their biocompatibility. The membranes were incubated with immortalized human keratinocytes (HaCaT) cultured in Dulbecco’s Modified Eagle Medium (DMEM) supplemented with 10% fetal bovine serum (FBS) and 1% Penicillin streptomycin and seeded at a density of 5x10^4^ cells/mL and volume of 90 μL/well in a 96-well plate. After 24 h of attachment, cells were treated for 48 h with 10 μL of the membrane solutions in triplicate, which resulted in final concentrations of 3.125, 12.5, 25, 50, 100, and 200 μg/mL. The negative control cells were treated with 1xPBS, while the positive control had 10% DMSO. To the 96-well plates, a solution of MTT was added and incubated for 4 h. Subsequently, formazan crystals were solubilized overnight with a solubilization reagent, and the absorbance values were measured at 570 nm [43]. The analysis was carried out in triplicate, and the % cell viability of all the membranes was compared to that of the untreated cells and was evaluated using Equation (5):

(5) $$\%\;Cell\;viability=\frac{A_{Mslm/dlm}-A_{z}}{A_{w}-A_{z}}\times 100\%$$

where **A_Mslm/dlm_** is the membrane’s absorbance, **A_z_** represents the absorbance of a blank, and **A_w_** is the absorbance of the untreated sample.

#### 2.3.12. In Vitro Whole Blood Assay

The whole blood assay was performed following the method reported in previous studies [44,45]. Selected membranes (2 mg) were immersed in 200 µL of whole blood to activate coagulation, and CaCl_2_ (20 µL) was added. The samples were incubated in a thermostatic incubator for 10 min with gentle shaking at 37 °C. To hemolyze the red blood cells (RBCs), approximately 6 mL of deionized water was added. The relative absorbance of the blood samples was measured at a wavelength of 540 nm after dilution to 25 mL.

#### 2.3.13. In Vitro Scratch Assay

Using a previously reported procedure, the in vitro scratch wound healing assay was performed on the membranes (SLMs and DLMs) [46,47,48]. HaCaT cells were utilized for this study. They were grown in a humidified incubator, maintaining a 37 °C temperature and a 5% CO2 level to reach 90% confluence, in DMEM enriched with 10% fetal bovine serum (FBS) and 1% Penstrep. The cells were trypsinized, and the number of viable cells was determined by excluding the trypan blue dye. The density of the cells was set to 2.5 × 10^5^ cells/mL, and they were seeded into 6-well plates, where a cell monolayer was observed to have formed after 48 h. On 6-well plates, each cell was inflicted with a scratch wound using a micropipette with a 200 µL tip. The wells were rinsed once or washed with 2 mL of 1× PBS to remove dislodged cells, and 1800 µL of serum-poor DMEM (DMEM with 1% FBS) was added to each well. After that, the cells were treated with 200 µL of various membrane concentrations to enhance the cell viability in the MTT assay. The cultured untreated cells in DMEM (with 10% FBS) acted as a positive control, while DMEM with 1% FBS was the negative control. To capture the scratch images, an inverted light microscope and 4× objective lens were employed, which had a phase contrast feature at 0, 24, 48, 72, and 96 h (Olympus CKX53, Tokyo, Japan). Scratch images were captured in triplicate, ImageJ software (ImageJ 1.53t) was used to measure cell migration, and wound closure (WC) was calculated using Equation (6):

(6) $$\mathrm{WC}\;\%=\frac{W_{A0h-W_{A96h}}}{WA0h}\times$$

***W_A0h_*** refers to the wound scratch area at the 0 h time interval, and ***W_A96h_*** represents the wound scratch area at the 96 h interval.

### 2.4. Plant Extract

The *Capparis sepiaria* root extract was analyzed using FTIR, ultraviolet–visible spectroscopy (UV-vis), Perkin Elmer Lambda 365 UV-Vis Spectrophotometer (Shelton, CT, USA), [M1] [AA2] and ultra-performance liquid chromatography–mass spectrometry (UPLC-MS) PDA detector Waters Acquity Ultra Performance Liquid Chromatographic System (Milford, MA, USA) [49].

### 2.5. Statistical Analysis

The data obtained from the in vitro studies were then evaluated using Student’s unpaired *t*-test. The obtained data are expressed as the mean ± standard deviation in triplicate (*n* = 3), and a *p*-value ≤ 0.05 is considered significant.

## 3. Results and Discussion

The UPLC-MS chromatograms of the *Capparis sepiaria* plant extract revealed the following phytochemical constituents: gluconic acid, citric acid, epicatechin, and Phloretin-2-o-glucoside with signals at 195.0500, 191.0208, 289.0389, and 435.1317, respectively [49]. Phytochemicals play significant roles in wound healing.

### 3.1. FTIR

**Figure 1:**
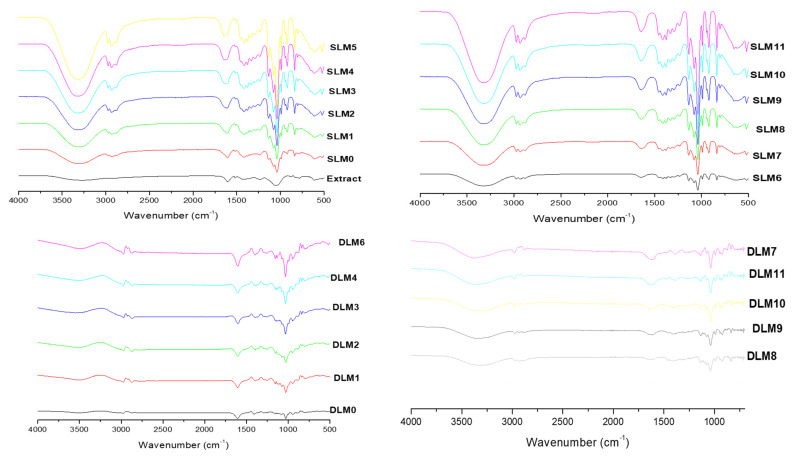
The FTIR spectra for the prepared SLMs and DLMs.

FTIR spectroscopy was used to determine the functional groups present in the membranes corresponding to the polymers (sodium alginate, PVA, gum arabic, and Pluronic F127) and *Capparis sepiaria* extract (gluconic acid, citric acid, epicatechin, and Phloretin-2-o-glucoside) used for their fabrication. The FTIR spectra of the membranes are shown in Figure 1. The SLMs displayed characteristic peaks of C-H stretching (2850–2960 cm^−1^), O-H stretching (3553 cm^−1^), C=O stretching (1680–1750 cm^−1^), C=C stretching (1640–1680 cm^−1^), and C-O stretching (1050–1150 cm^−1^) [25]. The FTIR spectra of the membranes showed significant peaks at 1032 cm^−1^, 1680 cm^−1^, 1644 cm^−1^, 2901 cm^−1^, and 3553 cm^−1,^ which overlapped with the C-O stretching of SA, PVA, and gum acacia, which are related to the C-O vibration stretching of PF127 [50]. In addition, the C-O, C=O, O-H, and C-H vibration stretching of SA, PVA, gum acacia, and PF127 overlapped, confirming their presence in the membrane. The SLMs displayed broader peaks of O-H stretch and C=O stretch when compared to the DLMs. However, the SLMs and DLMs displayed similar characteristic peaks. The FTIR spectra of the membranes, both loaded and non-loaded, demonstrated a band at 3726–2892 cm^−1^ due to the O–H and C-H stretching present in SA, PF127, PVA, and gum acacia.

### 3.2. SEM

**Figure 2:**
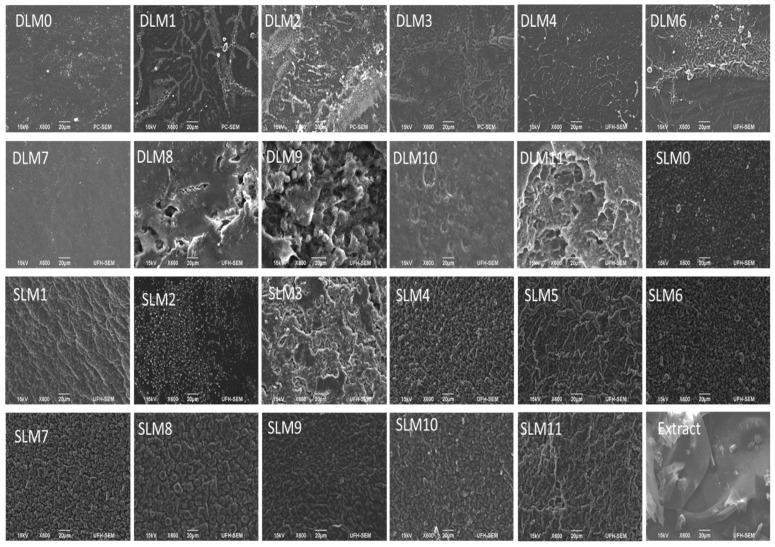
The SEM images for the prepared membranes and *Capparis sepiaria* extract.

SEM analysis was performed to evaluate the membranes’ surface morphology and their porous nature. The SEM images of the membranes are shown in Figure 2, displaying their surface morphologies. The SEM images of all DLMs displayed a smooth, woven, and irregular morphology. SLM 3, SLM10, and SLM11 exhibited plate-like and porous morphologies, whereas the other SLM displayed a sphere-like, globular, interconnected network with few micropores. Previous studies have reported similar morphologies [51,52,53,54]. The smooth and interconnected networks were attributed to sodium alginate. The porous structure of wound dressings is essential for good gaseous permeation that promotes cell proliferation, attachment, and nutrient migration, accelerating the wound-healing process. Furthermore, the porous morphology of the wound dressings also influences their water adsorption capacity [55,56].

### 3.3. AFM

Atomic force microscopy (AFM) was used to evaluate the DLM0 membrane to determine the surface roughness, as shown in Supplementary Figure S1. Surface roughness contributes to the membranes’ proliferation capability and cellular adhesion [57]. The surface roughness of the membrane was 126.83 ± 74.156 nm with a root mean square roughness of 160.67 ± 98.45 nm and a maximum roughness of 887.33 ± 1993.73 nm. The average surface roughness of the membrane is suitable for cell attachment/adhesion to promote skin regeneration [58].

### 3.4. pH, Porosity, and WVTR

The pH values of the prepared membranes are presented in Table 2. The pH values of the membranes were evaluated to determine their suitability for application in skin wound management. The pH of the membranes loaded with *Capparis sepiaria* ranged from 4.32 ± 0.03 to 8.00 ± 1.45, which was within the normal pH range of the skin. This pH is acceptable to avoid the risk of irritation upon application to the skin. The pHs of DLMs and SLMs were in the ranges of 6.22 ± 0.10–7.76 ± 0.83 and 4.32 ± 0.03–8.00 ± 1.45, respectively. The pH values of DLMs were closer to a neutral pH than SLMs, suggesting that the concentrations of the polymers used in the preparation of the DLMs contributed to their pH range. Najafi-Taher et al. [59] reported that wound dressings with pH values close to 7 are appropriate for dermal applications.

The % porosities of the prepared membranes are presented in Table 2. The % porosity of the membranes ranged from 11.50 ± 3.31 to 76.16 ± 46.71%. A similar porosity range was reported by both Ndlovu et al. and Buyana et al. for wound dressings [60,61]. The loading of *Capparis sepiaria* and the composition of the wound dressing improved the porosity of the membranes (SLM and DLM). The % porosity of the fabricated wound dressings increased when the concentration of the polymers was increased, as seen in the DLMs. DLM1 demonstrated the highest porosity of 76.16 ± 46.71%, while DLM9 displayed the lowest % porosity of 11.50 ± 3.31%. The polymer composition significantly affected the porosity of the prepared membranes. Ngece et al. [62] reported that an increase in the concentration of biopolymers used for the wound dressing formulation significantly affected the % porosity of the wound dressings. Most of the reported sodium alginate-based wound dressings exhibited optimal porosity, which is useful for gaseous diffusion and the migration of nutrients to the wound bed, permitting the exchange of substances between the cells of the skin, promoting the absorption of wound exudates, and stimulating high cell adhesion and proliferation, thereby accelerating wound healing.

WVTR is one essential property of wound dressings. It provides information on a wound dressing’s ability to control exudate retention and absorption from the wound bed [61]. The WVTR values of the prepared membranes (SLMs and DLMs) are shown in Table 2. The WVTR of the single- and double-layer membranes ranged from 81.22 ± 0.73 to 1654.73 ± 0.74 g/m^2^/day, while the commercially available wound dressing demonstrated a WVTR of 81.73 ± 51.43. The prepared membranes displayed a higher WVTR than the commercial wound dressing. Increasing the concentration of the polymers in the DLMs significantly enhanced their WVTR when compared to SLMs. The interwoven morphology of the wound dressing and low % porosity contributed to their low WVTR. Factors such as the thickness, porosity, and chemical properties of the materials used to design wound dressings influence the WVTR. It is important to note that some commercially available wound dressings, such as Dermiflex^®^ and J&J, have been reported with a WVTR of 90 g/m^2^/day [63]. The WVTR range suitable for wound dressing should not exceed 2000 to 2500 g/m^2^/day [63,64,65]. The highest WVTR was 1654.73 ± 0.74 g/m^2^/day, which is close to the ideal range for WVTRs, indicating that they can prevent moisture accumulation, dehydration, exudate accumulation, and reduce the risk of infection. Membranes with higher SA content displayed increased WVTR values.

### 3.5. Swelling Behavior

The swelling behavior of the prepared wound dressings was evaluated to observe their ability to absorb exudates, as shown in Table 3. The ability of a wound dressing to absorb exudates plays a significant role in maintaining a moist environment [66]. The swelling behavior of the prepared membranes also plays a crucial role in the release rate of the loaded drug in the membrane and the biological activity. A slow swelling ratio delays the release of the loaded drug and vice versa. Good swelling behavior aids adhesion, optimum moisture content, and wound exudate absorption, stimulating keratinocyte migration and fibroblast proliferation [66]. The DLMs demonstrated swelling ratios ranging from 37 to 169%. Some membranes reached a maximum swelling after 1, 3, and 24 h, respectively. DLM0, DLM1, and DLM4 displayed maximum swelling over 24 h. DLM3’s swelling capability was the highest over 3 h. Due to the high solubility of the SLMs at pH 5.5 and 7.4, they were not evaluated for their swelling behavior. Other researchers have reported similar findings for wound dressings with good swelling capability [67,68]. The swelling behavior of the membranes is a critical parameter that reflects their ability to absorb exudates from the wound site. This feature is essential for maintaining a moist environment that is suitable for accelerated healing while preventing the excessive accumulation of wound fluids, which can lead to maceration of the surrounding skin [69]. Incorporating plant extracts into membranes could enhance their healing property [70].

### 3.6. XRD

**Figure 3:**
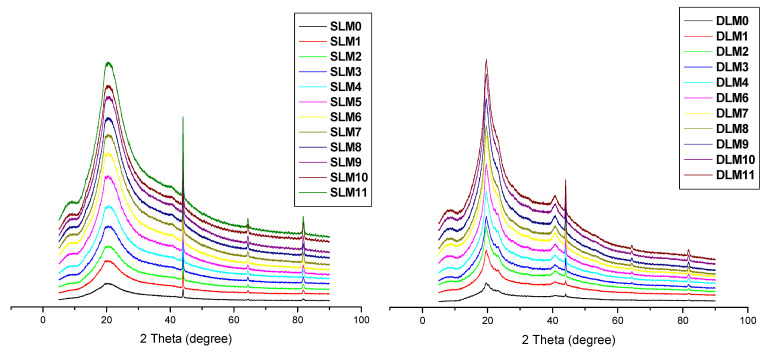
XRD for the prepared SLMs and DLMs.

XRD analysis was performed on the membranes to evaluate their physical nature (i.e., crystalline or amorphous), as shown in Figure 3. The membrane displayed broad peaks, indicating an amorphous nature [71,72,73]. However, a sharp peak indicating a semicrystalline nature was visible on the single- and double-layer membranes at 2Ɵ = 44°. The broad and weak diffraction peaks result from strong intramolecular and intermolecular hydrogen bonding between the polymer chains formed from the crosslinking process [74,75,76]. Amorphous wound dressings offer the advantage of maintaining a moist healing environment and conforming to the wound bed, which can enhance the healing process [77].

### 3.7. Biodegradation Studies

The degradability of the prepared membranes was studied using pH-simulating wound exudates (Table 4). After one week of study, the membranes showed weight loss ranging from 56 to 71%, and after three weeks, the weight loss ranged from 55 to 69% less than in the first week of degradation, as shown in Table 4. This indicates that the degradation of the prepared membranes was rapid in the first week but moderate in the third week, which might be due to the polymer composition employed for the preparation of the wound dressing [39]. Biodegradation occurs due to the swollen state of the membranes, followed by hydration and bond cleavage. The degradability of the prepared wound dressings indicates their suitability for skin regeneration. The biodegradable nature of the wound dressings suggests that they will be rapidly absorbed into the newly formed tissue to promote smooth tissue regeneration without trauma and scar formation [78].

### 3.8. Mechanical Properties

The physical characteristics of the membranes were analyzed for their tensile strength and Young’s modulus. The tensile strength is the maximum force per unit area applied to the point at which the object breaks [79]. The tensile strength of the membranes ranged from 0.1703 ± 0.353 to 52.559 ± 20.979 MPa, which is suitable for the skin. The membranes with the lowest tensile strengths were the SLMs, as shown in Table 5. The range of tensile strength that has been reported to be ideal for a wound dressing is between 1 and 32 MPa [80]. Tensile strength is a critical parameter because it reflects the ability of the dressing to resist breaking under tension. It influences the flexibility of wound dressings. High-tensile strength wound dressings are characterized by high flexibility, which allows such wound dressings to adapt to folding areas and also provide ease of movement to the injury patients [81]. Legon’Kova et al. emphasized the importance of understanding the operational properties of wound dressings, including their tensile strength, to ensure their comfort and ease of use [69]. The tensile yield strain indicated that wound dressings should have sufficient flexibility to conform to body contours without breaking, implying that a certain degree of yield strain is necessary, as shown in Table 5. The elastic modulus or Young’s modulus measures the stiffness of a wound dressing [81]. The SLMs exhibited no elastic modulus, indicating their high degree of softness. The Young’s modulus of the DLMs ranged from 21.085 ± 78.291 MPa to 115.6967 ± 53.512 MPa, indicating that the wound dressing was elastic and could be easily stretched, which is crucial for the comfort and functionality of the prepared dressing.

Similar tensile strengths were reported by Simões et al. [82] for membranes developed for treating skin injuries. The tensile strengths were 3.43 ± 0.82 MPa, 13.16 ± 1.68 MPa, 13.55 ± 3.89 MPa, 4.41 ± 0.89 MPa, 5.67 ± 0.01 MPa, and 7.80 ± 2.98 MPa, which are similar to those displayed by the native skin tensile strength in the range of 5.00–30.00 MPa [83].

### 3.9. In Vitro Antibacterial Analysis

Antibacterial studies were performed on the plant extract, control (commercial wound dressing), and membranes (SLM and DLM) encapsulated with *Capparis sepiaria* plant extract to evaluate the antimicrobial efficacy of the wound dressings. The antibacterial efficacy of the membranes against Gram-negative and Gram-positive bacterial strains was observed by comparing their minimum inhibition concentration (MIC) values to those of the references (controls, plant extract, AMP, NLD, and STM), as shown in Table 6. The lowest MIC values of the membranes were more effective than those reported in the literature [84]. The antibacterial activity of the plant extracts encapsulated in the SLMs was retained. The loading of *Capparis sepiaria* into the SLMs did not improve the antibacterial efficacy. Only SLM3, SLM5, and SLM11 demonstrated antibacterial efficacy against bacterial strains compared to other SLMs. The SLM5 membrane was effective against bacterial strains such as *Pseudomonas aeruginosa*, *Proteus vulgaris*, *Enterococcus faecalis*, *Enterobacter cloacae*, and *Escherichia coli*, which have been reported to contribute to wounds being chronic [85,86,87]. DLMs showed enhanced antibacterial efficacy compared with SLMs. The double chemical composition of the DLMs improved their antibacterial efficacy. All the DLMs displayed excellent antibacterial efficacy against both Gram-negative and Gram-positive bacteria. *Pseudomonas aeruginosa*, *Enterobacter cloacae*, *Enterococcus faecalis*, *Enterobacter cloacae*, *Mycobacterium smegmatis*, *Escherichia coli*, and *Proteus vulgaris* are responsible for infections that prolong wound healing. The DLMs demonstrated enhanced antibacterial activity, which is attributed to their bilayer structure. Similar findings have been reported for bilayer-structured wound dressings with enhanced antibacterial activity [87]. These membranes are promising wound dressing owing to their antimicrobial properties.

### 3.10. In Vitro Cell Viability

**Figure 4:**
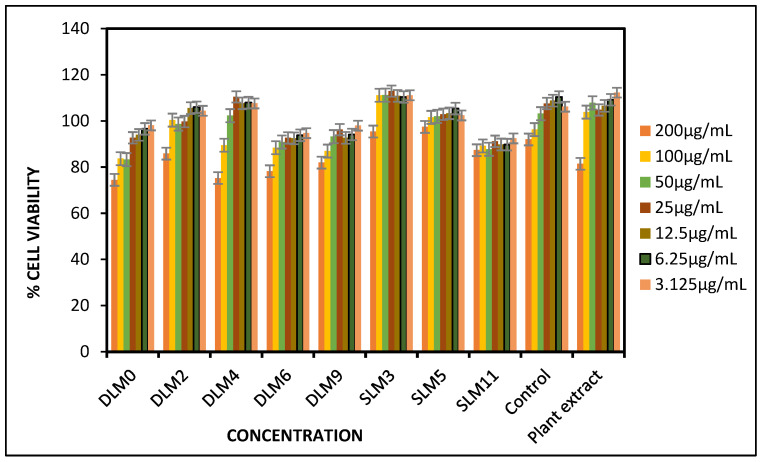
Cell viability of HaCaT cells treated with the selected membranes (DLM0, DLM2, DLM4, DLM6, DLM9, SLM3, SLM5, and SLM11), control (commercial wound dressing), and plant extract after 48 h; testing was conducted using an MTT assay at a wavelength of 570 nm.

In vitro cytotoxicity was assessed on the plant extract, control (Burn-EAZ), and selected membranes that showed enhanced antibacterial efficacy incorporated with the *Capparis sepiaria* root extract by screening them at concentrations of 3.125, 6.25, 12.2, 25, 50, 100, and 200 μg/mL against HaCaT cells to evaluate their biocompatibility, as shown in Figure 4. The cell viability of the membranes was calculated against untreated cells to analyze their cytotoxicity. The plant extract exhibited the highest % cell viability when compared to the prepared wound dressings and control, as shown in Figure 4, suggesting that the presence of the *Capparis sepiaria* extract enhanced the biocompatibility of the membrane dressing by promoting cell migration and proliferation. This could be due to the ability of the extracts to create a favorable microenvironment for cell survival [39,88]. The prepared membranes were not toxic to the HaCaT cells and had excellent % cell viability of 94% or higher. The SLM3 membranes induced the highest cell viability, ranging from 96 to 111%, at all the concentrations used in the study. SLM3 exhibited good cell viability, indicating non-toxicity and excellent biocompatibility, which is an ideal feature of an effective wound dressing. The membranes demonstrated excellent cell viability of at least 94% at the lowest concentration of 3.125 μg/mL, and a % cell viability of 75% and above was observed at the highest concentration of 200 μg/mL. The membranes displayed excellent biocompatibility [89]. Sodium alginate’s non-toxic properties contributed to the enhanced biocompatibility of the membranes [90]. The combination of *Capparis sepiaria* extract and sodium alginate resulted in the enhanced biocompatible nature of the membranes, highlighting the efficacy of SA and plant-based bioactive agents in the design of wound dressings.

### 3.11. In Vitro Hemostasis Analysis

**Figure 5:**
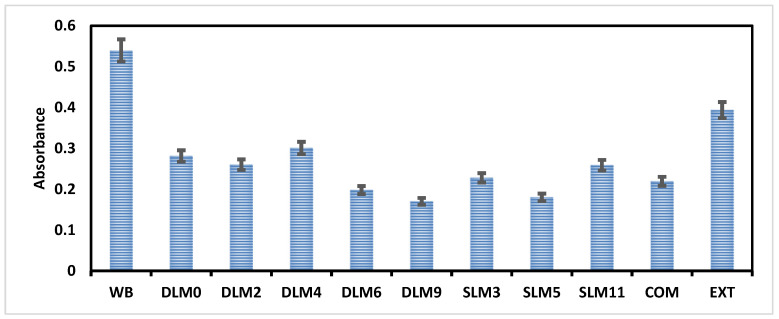
The absorbances of the membranes (DLM0, DLM2. DLM4, DLM6, DLM9, SLM3, SLM5, and SLM11), commercial wound dressing (COM), and *Capparis sepiaria* plant extract (EXT) were compared to whole blood (WB) at 540 nm, i.e., *p* < 0.0001–0.0018, with a 95% confidence interval.

The analysis was performed on *Capparis sepiaria* plant extract, the selected membranes that displayed enhanced antibacterial efficacy (i.e., DLM0, DLM2, DLM4, DLM6, DLM9 SLM3, SLM5, and SLM11), COM (commercial wound dressing), and whole blood (WB). The selected membranes were compared with WB to observe their ability to control bleeding, as shown in Figure 5. The selected membranes and COM demonstrated an absorbance less than the WB and plant extracts, with absorbances ranging from 0.17 to 0.30, suggesting that all the prepared membranes can induce blood clotting. DLM9 displayed the lowest absorbance value followed by SLM5, DLM6, COM, SLM3, SLM11, DLM2, DLM0, and DLM4. DLM9, SLM5, and DLM6 revealed a high clotting ability compared to the commercially available wound dressings. SLM3 and COM showed absorbance values of 0.22 and 0.23, respectively, indicating similar clotting abilities. Sodium alginate and *Capparis sepiaria* extracts have been reported to have hemostatic effects [25,90,91]. The *Capparis sepiaria* plant extract, when combined with sodium alginate, demonstrated an enhanced synergistic hemostatic effect. Hemostasis is the first phase of the wound-healing process and, if not controlled, can lead to delayed wound healing because it plays a major role in the inflammatory phase [24,92]. PVA has also been reported to enhance the hemostatic effect [60]. The polymer composition and quantity employed for the fabrication of the membranes contributed to their good hemostatic efficacy, and similar findings were reported by researchers for wound dressings that promoted good clotting capability [93,94]. The membranes showed promising hemostatic effects, making them potential wound dressings for managing bleeding wounds.

### 3.12. In Vitro Scratch Wound Healing Assay

**Figure 6:**
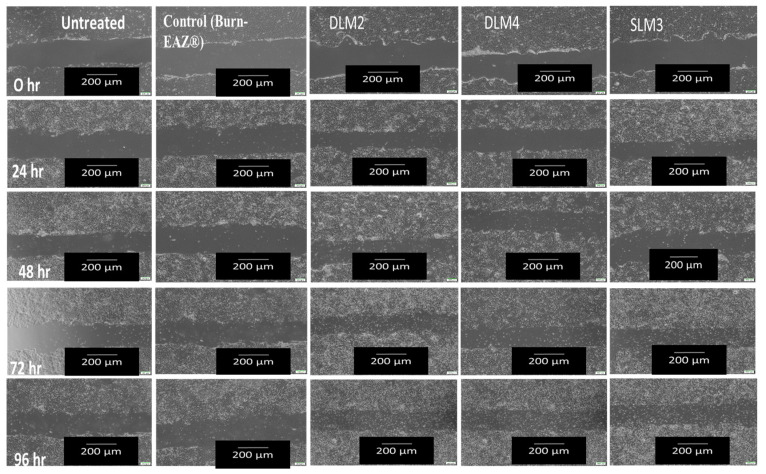
The images of membranes (DLM2, DLM4, and SLM3), the control, and untreated cells for the wound healing scratch assay.

An in vitro scratch wound healing assay was conducted on selected membranes (i.e., DLM2, DLM4, and SLM3), which were loaded with *Capparis sepiaria* root extract, which showed the highest % cell viability compared to the other prepared membranes (Figure 6). The wound healing assay was conducted for four days at time intervals of 0, 24, 48, 72, and 96 h to compare the rate of wound closure on untreated cells, cells treated with the prepared membranes, and cells treated with the control (commercial wound dressing); the data are shown in Figure 6. The selected wound dressings exhibited a higher rate of wound closure than the untreated cells and cells treated with commercially available wound dressings, indicating that the prepared wound dressings are promising materials that will promote rapid wound healing. SLM3 demonstrated the highest rate of wound closure compared with cells treated with DLM2 and DLM4 after 96 h. SLM3-, DLM2-, and DLM4- treated cells exhibited a reduction in the wound scratch area with a rate of wound closure of 18%, 48%, and 68%, respectively, after four days. DLM demonstrated enhanced antibacterial activity, biocompatibility, and hemostatic effect. Meanwhile, after four days, the untreated cells and those treated with the control demonstrated closure rates of 17% and 21%, respectively. The double-layer-based membranes supported cellular adhesion and migration significantly more than the single-layer membranes. Double-layer membranes have been reported for enhanced biological studies [95], and they offer enhanced properties, such as increased porosity and adsorption capacity [22,23]. The *Capparis sepiaria* root extract and the polymers’ non-toxic effect also contributed to the membranes supporting the migration of HaCaT cells, which is a crucial feature for accelerated wound healing.

## 4. Conclusions

Novel sodium alginate-based single- and double-layer membrane wound dressings were fabricated using a solvent-casting method in this study. The prepared wound dressings were further incorporated with a *Capparis sepiaria* root extract to enhance their properties, such as antimicrobial efficacy, clotting capability, and wound-healing effect. The prepared membranes demonstrated a porous and amorphous nature, suitable WVTR, exudate absorption capability, and blood clotting capability. These membranes are biodegradable and non-toxic to human skin cells. The double-layer membranes demonstrated enhanced properties in all analyses compared with the single-layer membranes. DLM9, DLM2, and DLM4 demonstrated the highest antibacterial activities when compared to all the prepared membranes. The membrane with the highest WVTR was DLM4, while the lowest was SLM6. The membrane that revealed the lowest absorbance, revealing the highest blood clotting capability, was DLM9, with an absorbance of 0.17. The membranes were effective against various bacterial strains responsible for wound infections. All the results obtained from the research showed that the membranes prepared are ideal for use as wound dressings, and further studies would be necessary to fully understand their mechanism of action. The membranes DLM 2, DLM4, DLM9, SLM3, and SLM5 are promising wound dressings that require further evaluation.

## Figures and Tables

**Table 1:** (a) Composition of the prepared SLMs loaded with *Capparis sepiaria* plant extract. (b) Composition of the prepared DLMs loaded with *Capparis sepiaria* plant extract.

**(a)**							
**Sample Code**	**SA (mg)**	**Gum Acacia (mg)**	**PF127 (mg)**	**PVA (mg)**	**Propylene Glycol (mL)**	**DH_2_O (mL)**	***Capparis sepiaria* Extract (mg)**
SLM0	400	-	300	500	4	5	-
SLM1	400	-	300	500	4	5	100
SLM2	100	300	300	500	4	5	100
SLM3	200	200	300	500	4	5	100
SLM4	300	100	300	500	4	5	100
SLM5	-	400	300	500	4	5	100
SLM6	500	-	300	400	4	5	100
SLM7	600	-	300	300	4	5	100
SLM8	500	-	300	200	4	5	100
SLM9	400	200	300	300	4	5	100
SLM10	300	200	300	400	4	5	100
SLM11	300	300	300	300	4	5	100
**(b)**							
**Sample Code**	**SA (mg)**	**Gum Acacia (mg)**	**PF127 (mg)**	**PVA (mg)**	**Propylene Glycol (mL)**	**DH_2_O (mL)**	***Capparis sepiaria* Extract (mg)**
DLM 0	800	-	600	1000	4	10	-
DLM 1	800	-	600	1000	4	10	100
DLM 2	200	600	600	1000	4	10	100
DLM 3	400	400	600	1000	4	10	100
DLM 4	600	200	600	1000	4	10	100
DLM 5	-	800	600	1000	4	10	100
DLM 6	1000	-	600	800	4	10	100
DLM 7	1200	-	600	600	4	10	100
DLM 8	1000	-	600	400	4	10	100
DLM 9	800	400	600	600	4	10	100
DLM 10	600	400	600	800	4	10	100
DLM 11	600	600	600	600	4	10	100

**Table 2:** The surface pH, % porosity, and WVTR of the fabricated membrane wound dressing compared to the control.

Sample Code	pH	% Porosity	WVTR (g/m^2^/day)
SLM0	6.75 ± 0.08	19.27 ± 3.64	168.57 ± 143.88
SLM1	6.83 ± 0.39	54.74 ± 7.72	137.29 ± 0.43
SLM2	6.42 ± 0.15	26.60 ± 1.70	279.96 ± 0.24
SLM3	6.54 ± 0.12	13.56 ± 5.63	127.35 ± 0.013
SLM4	5.26 ± 0.12	29.70 ± 3.00	85.03 ± 2.54
SLM5	4.32 ± 0.03	21.84 ± 2.29	119.06 ± 1.934
SLM6	5.52 ± 0.14	26.36 ± 2.12	81.22 ± 0.73
SLM7	5.81 ± 0.13	25.85 ± 0.89	166.75 ± 0.73
SLM8	8.00 ± 1.45	22.31 ± 10.36	92.98 ± 0
SLM9	5.20 ± 0.09	28.41 ± 0.34	138.46 ± 2.64
SLM10	5.15 ± 0.07	17.05 ± 1.79	145.86 ± 5.13
SLM11	5.81 ± 0.13	22.13 ± 3.85	87.85 ± 14.86
DLM 0	7.76 ± 0.83	22.77 ± 2.18	974.64 ± 2.20
DLM 1	6.34 ± 0.12	76.16 ± 6.71	929.30 ± 30.11
DLM 2	6.30 ± 0.03	48.33 ± 0.08	454.33 ± 0.73
DLM 3	6.89 ± 0.29	29.53 ± 7.28	694.68 ± 4.57
DLM 4	6.26 ± 0.03	24.89 ± 5.47	1654.73 ± 0.74
DLM 6	6.22 ± 0.10	29.37 ± 1.93	1308.14 ± 0.72
DLM 7	6.93 ± 0.28	29.40 ± 30.07	1069.45 ± 8.63
DLM 8	6.48 ± 0.04	29.59 ± 10.50	1201.56 ± 12.71
DLM 9	6.32 ± 0.19	11.50 ± 3.31	1244.32 ± 11.44
DLM 10	6.43 ± 0.12	31.78 ± 1.37	729.32 ± 19.37
DLM 11	6.50 ± 0.11	25.15 ± 5.17	457.48 ± 10.84
Control (commercial wound dressing)	-	-	81.73 ± 51.43

**Table 3:** Swelling behavior of the membranes.

Sample Code	1 h	3 h	24 h
DLM 0	142.11 ± 3.14	115.03 ± 17.71	166.09 ± 4.90
DLM 1	80.62 ± 3.24	77.16 ± 4.27	113.53 ± 0.44
DLM 2	131.45 ± 0.86	125.49 ± 13.16	-
DLM 3	155.43 ± 10.89	196.69 ± 10.20	-
DLM 4	87.70 ± 15.73	72.85 ± 10.62	102.13 ± 12.19
DLM 6	37.50 ± 2.16	46.43 ± 3.74	-
DLM 7	51.21 ± 4.28	55.41 ± 5.16	-
DLM 8	78.29 ± 3.27	92.90 ± 3.27	75.92 ± 5.24
DLM 9	141.28 ± 3.11	163.10 ± 4.18	-
DLM 10	166.15 ± 15.87	140.67 ± 20.76	-
DLM 11	169.43 ± 2.32	167.0 ± 66.30	-

**Table 4:** Results of biodegradation studies for selected membranes at pH 7.4.

Sample Code	Biodegradation		
	W1	W2	W3
DLM0	63.63 ± 0.74	57.46 ± 1.41	60.62 ± 0.31
DLM1	56.60 ± 0.67	48.46 ± 0.80	55.14 ± 0.45
DLM3	70.74 ± 0.72	68.61 ± 8.56	69.51 ± 3.87
DLM6	71.41 ± 0.49	68.63 ± 0.31	67.75 ± 0.82

**Table 5:** Mechanical properties of the membranes for tensile strength and Young’s modulus.

Sample Code	Maximum Load (N)	Tensile Yield Strength (MPa)	Elastic Modulus (MPa)	Tensile Yield Strain (%)
SLM0	0.24 ± 0.764	0.19 ± 0.56	-	17.14 ± 12.92
SLM3	0.22 ± 0.30	0.17 ± 0.35	-	10.35 ± 10.05
SLM5	0.29 ± 0.03	0.21 ± 0.03	-	61.22 ± 32.12
DLM0	73.54 ± 29.33	52.56 ± 21.00	115.70 ± 53.51	248.78 ± 137.43
DLM2	13.86 ± 23.84	9.93 ± 17.03	-	39.78 ± 22.09
DLM4	28.57 ± 18.93	16.90 ± 6.33	43.74 ± 51.46	194.70 ± 425.71
DLM6	44.35 ± 17.71	31.75 ± 10.11	77.03 ± 104.32	280.46 ± 463.12
DLM7	22.99 ± 44.03	15.08 ± 33.01	108.88 ± 00	76.70 ± 144.61
DLM8	31.14 ± 14.13	22.25 ± 10.11	21.09 ± 78.29	361.07 ± 23.40
DLM9	27.01 ± 13.08	12.29 ± 4.25	49.53 ± 62.11	71.93 ± 151.84
DLM11	12.59 ± 14.32	8.43 ± 12.43	-	289.37 ± 625.52

**Table 6:** Antimicrobial efficacies of the DLMs and SLMs against Gram-positive and Gram-negative strains of bacteria.

Minimum Inhibitory Concentration (MIC, µg/mL)												
Test Compound	Gram Positive					Gram Negative						
	BS	EF	SE	SA	MS	ECL	PV	KO	PA	PM	EC	KA
DLM1	15.625	15.625	-	15.625	15.625	15.625	15.625	15.625	15.625	500	500	500
DLM0	500	500	-	500	500	500	500	500	500	500	500	500
DLM2	15.625	15.625	-	500	15.625	15.625	15.625	15.625	15.625	15.625	15.625	500
DLM3	15.625	15.625	-	500	15.625	15.625	15.625	500	15.625	500	500	500
DLM4	500	15.625	-	500	500	15.625	15.625	15.625	15.625	15.625	15.625	15.625
DLM6	500	15.625	-	500	500	15.625	15.625	15.625	15.625	500	15.625	500
DLM7	500	15.625	-	500	15.625	15.625	500	15.625	15.625	500	15.625	500
DLM8	500	500	-	500	500	500	500	500	500	500	500	500
DLM9	15.625	15.625	500	500	15.625	15.625	15.625	15.625	15.625	15.625	15.625	15.625
DLM11	500	15.625	500	500	500	15.625	15.625	500	15.625	500	500	500
SLM0	500	500	500	500	500	500	500	500	500	500	500	500
SLM3	500	500	500	500	500	500	500	15.625	15.625	15.625	500	500
SLM5	500	15.625	500	500	500	15.625	15.625	500	15.625	15.625	15.625	125
SLM11	500	500	500	500	500	500	500	15.625	500	15.625	15.625	15.625
EXT CNT	15.625 15.625	15.625 500	500 500	15.625 15.625	500 500	15.625 15.625	15.625 15.625	500 500	15.625 15.625	500 15.625	31.25 15.625	500 15.625
AMP	26	26	26	26	26	26	416	26	64	26	26	26
STM	16	128	8	256	4	512	128	16	128	128	64	512
NLD	16	>512	64	64	512	16	128	8	128	32	512	256

*Bacillus subtilis* (BS), *Enterococcus faecalis* (EF), *Mycobacterium smegmatis* (MS), *Staphylococcus epidermidis* (SE), *Escherichia coli* (EC), *Enterobacter cloacae* (EC), *Klebsiella oxytoca* (KO), *Proteus vulgaris* (PV), *Pseudomonas aeruginosa* (PA), *Proteus mirabilis* (PM), *Staphylococcus aureus* (SA), *Klebsiella pneumonia* (KP), *Capparis sepiaria* root extract (EXT), Burn-EAZ (CNT, commercial wound dressing), Streptomycin (STM), and nalidixic acid (NLD).

## Data Availability

The original contributions presented in the study are included in the article/Supplementary Material, further inquiries can be directed to the corresponding author/s.

## Supplementary Materials

The following supporting information can be downloaded at: https://www.mdpi.com/article/10.3390/pharmaceutics16101313/s1, Figure S1: AFM image of DM0.
