# Supplementary material for: Capparis sepiaria-Loaded Sodium Alginate Single- and Double-Layer Membrane Composites for Wound Healing
Source: Pharmaceutics. 2024 Oct 10;16(10):1313. doi: 10.3390/pharmaceutics16101313 (PMC11510319; doi:10.3390/pharmaceutics16101313)
Supplement: Supplementary file 1 [file pharmaceutics-16-01313-s001.zip › pharmaceutics-3177081-supplementary.pdf]

pharmaceutics

MDPI

Supplementary Material

Capparis sepiaria-Loaded Sodium Alginate Single and Double Layer Membrane Composite for Wound Healing

Sindi P. Ndlovu[1], Keolebogile S.C.M. Motaung[2], 'Mapula Razwinani[3], Sibusiso Alven[4], Samson A. Adeyemi[5], Philemon N. Ubanako[5], Lindokuhle M. Ngema[5], Thierry Y. Fonkui[6], Derek T. Ndinteh[7], Pradeep Kumar[5], Yahya E. Choonara[5], and Blessing A. Aderibigbe[1]*[1]

1 Department of Chemistry, University of Fort Hare, Alice Campus, Alice 5700, South Africa; 201304407@ufh.ac.za
2 Global Health Biotech Pty Ltd., Pretoria, South Africa; keo@globalhealthbiotech.co.za
3 Department of Biotechnology and Food Science, Faculty of Applied Sciences, Durban University of Technology, Durban 4000, South Africa; nomphar@yahoo.com
4 Department of Chemistry, Nelson Mandela University, 6001 South Africa; s217616712@mandela.ac.za
5 Wits Advanced Drug Delivery Platform Research Unit, Department of Pharmacy and Pharmacology, School of Therapeutic Science, Faculty of Health Sciences, University of the Witwatersrand, Johannesburg, South Africa; samson.adeyemi@wits.ac.za (S.A.A.); philemon.ubanako@wits.ac.za (P.N.U.); 845407@students.wits.ac.za (L.M.N.); pradeep.kumar@wits.ac.za (P.K.); yahya.choonara@wits.ac.za (Y.E.C.)
6 Department of Biotechnology and Food Technology, Faculty of Science, University of Johannesburg, Doornfontein Campus, Johannesburg 2028, South Africa; youmbifonkui@yahoo.com
7 Drug Discovery and Smart Molecules Research Labs, Centre for Natural Product Research, Department of Chemical Sciences, University of Johannesburg, Doornfontein Campus, Johannesburg, South Africa; dndinteh@uj.ac.za
* Correspondence: blessingaderibigbe@gmail.com or baderibigbe@ufh.ac.za

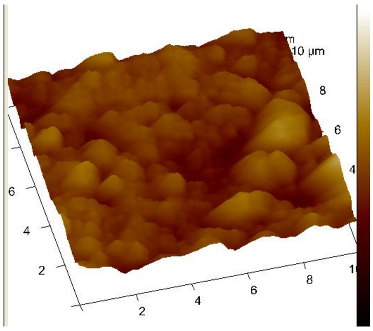

Figure S1: AFM image of DM0
